# A study of elastico-viscous fluid flow by a revolving disk with heat dissipation effects using HAM based package BVPh 2.0

**DOI:** 10.1038/s41598-021-83864-z

**Published:** 2021-02-25

**Authors:** M. Burhan Jafeer, M. Mustafa

**Affiliations:** School of Natural Sciences (SNS), National University of Sciences and Technology (NUST), Islamabad, 44000 Pakistan

**Keywords:** Applied mathematics, Computational science

## Abstract

Von Kármán problem of infinite disk is re-examined when fluid under consideration is elastico-viscous, satisfying the constitutive relations of Walters-B model. Main target here is to demonstrate how the presence of elasticity alters heat transfer phenomenon for the said problem especially when heat dissipation term is included in the analysis. We assume a self-similarity solution that results in a system of coupled non-linear equations. An easy to use package BVPh 2.0 based on the homotopy analysis method is used to present series solutions for values of elastico-viscous fluid parameter ($K$) in the range $0 \le K \le 1$. Residuals are evaluated numerically at various order of approximations which depict that obtained solutions converge to the exact solutions. Boundary layer is substantially suppressed due to the consideration of elastico-viscous fluid assumption. Furthermore, velocity of the entrained fluid is inversely proportional to the parameter $K$. The results predict a substantial drop in heat transfer rate whenever elasticity effects are present. A considerable role of heat dissipation towards thickening of thermal boundary layer is apparent from the findings.

## Introduction

Research interest in fluid motion triggered by the action of revolving disk has been ever growing since its discovery by Von Kármán^1^. Such an interest is attributed to its occurrence in some technological processes including electrochemistry (which involves rotating-disk electrodes), cooling of computer storage devices and food processing industries. Dynamics of rotating flows, their fundamental equations and practical applications were summarized in a book by Childs^2^. An excellent monograph was published by Shevchuk^3^ which includes formulation of convective heat transfer in various rotating flow configurations. Integral methods, self-similar techniques and analytical approaches to deal with rotating disk were also described briefly in this monograph. In the past, many novel results associated with the Von Kármán system have been introduced. We summarize here only a few (significant) problems for the sake of brevity. Stuart^4^ modeled the suction/blowing effects in rotating-disk system and found that radial and circumferential flows are decelerated whenever notion of suction is present. Heat transfer process in Von Kármán’s model was assessed in early studies (Refs.^5,6^). In^6^, by assuming a linear relationship between viscosity and temperature, the authors obtained series solutions for both small and large Prandtl numbers. Benton^7^ introduced time-dependency in Von Kármán’s model by assuming that disk rotation sets up impulsively from rest. Also, more accurate solution for steady-state case was found. Later, Watson and Wang^8^ considered more realistic model in which disk angular velocity was assumed to decay as time from the initiation of motion progresses. They concluded that the disk can be made stress free by assigning an appropriate value to a parameter measuring the disk rotation rate. Miclavcic and Wang^9^ examined Von Kármán flow when the boundary (or rotating disk) admits partial slip characteristic. It was noticed that velocity slip contributes to a growth in minimum torque needed to keep disk in steady rotation. In articles^10,11^, Shevchuk considered a power-law surface temperature distribution (proportional to $r^{n}$, where $r$ is the radial and $n$ is power-law index). He noticed that heat transfer rate of the infinite disk increases with power law index $n$ in case of positive radial temperature gradient while opposite trend is found for negative radial temperature gradient. Similar model and conclusions were also depicted in another paper by Shevchuk and Buschmann^12^. Xu and Liao^13^ put forward a novel analytic solution for the unsteady flow driven by a disk which undergoes impulsive rotation from rest. Later, Fang and Tao^14^ modeled fluid flow by a stretching and revolving disk with deceleration. Their analysis depicts that resisting wall shear vanishes by selecting a suitable ratio of disk stretching rate to the rotation rate. Von Kármán flow was also examined by Turkyilmazoglu^15^ when the disk was assumed to shrink in the radial direction. It was found that structure of solutions in shrinking case widely differ from those obtained in usual stretching case. Thermal transport in nanofluid flow triggered by a revolving disk was elucidated by Khan et al.^16^ by assuming a realistic zero mass flux condition. Muthtamilselvan and Renuka^17^ analyzed flow situation between rotating and stretchable disks in nanofluid. Application of a non-Fourier heat flux theory for micropolar fluid flow occurring between revolving surfaces was presented by Doh et al.^18^. A rigorous analysis for swirling flow between parallel disks, one undergoing uniform rotation and other stretching/shrinking, was made by Abbas et al.^19^. A novel model of homogeneous-heterogeneous reaction for nanofluid flow triggered between revolving disks was analyzed by Renuka et al.^20^. Using homotopy analysis method, entropy growth in nanofluid flow contained between spinning stretchable surface was elucidated by Renuka et al.^21^.

The frequent and broad occurrence of non-Newtonian behavior in diverse applications (both in nature and technology) is well established. Hoyt^22^ briefly summarized how non-Newtonian fluid flow is beneficial in some industrial processes including fluid friction reduction, surfactant applications for cooling/heating of large buildings and use of polymer additives to improve flow in petroleum pipe lines. Non-Newtonian behavior is also met in mining industry which treats slurries and muds, and in applications such as lubrication and biomedical flows. In most of the industrial processes, the Newtonian fluid assumption stands invalid and a complex non-Newtonian response needs to be modeled. In the past, various contributions featuring non-Newtonian fluid flow occurring above a disk that undergoes uniform rotation have been reported. The first ever attempt was made by Elliot^23^ who revisited Von-Karman’s analysis by taking into account constitutive relations due to Walters-B model. Decades ago, Ariel^24^ used a robust approach to describe the viscoelastic fluid flow due to revolving disk placed in a second-grade fluid. He was able to construct approximate series solutions for small and large values of second grade fluid parameter. Andersson and De Korte^25^ numerically addressed axial magnetic field effects on rotating disk induced flow of power-law fluid. Their solution was based on generalized von Kármán transformations, which was valid even for highly shear-thickening fluids. Ariel^26^ later revisited Elliot’s work with a view to obtain accurate numerical results for large elasticity fluid parameter. Von Kármán flow analysis for viscoplastic fluid was made by Osalusi et al.^27^ using the well-accepted Bingham model. The articles published by Attia^28^ and Sahoo ^29^ examined different physical characteristics associated with Reiner-Rivlin fluid flow caused by a rotating disk. Other contributions put forward in this domain include the works of Ahmadpour and Sadeghy^30^, Griffiths^31^, Guha and Sengupta^32^, Doh and Muthtamilselvan^33^, Tabassum and Mustafa^34^, Imtiaz et al.^35^, Sahoo and Shevchuk^36^ and Mustafa et al.^37^.

Our foremost interest is to formulate heat transfer in Von Kármán of Walters-B fluid under viscous dissipation effect. It will be shown later, that viscous dissipation effect yields several additional terms in the energy equation. Notably, viscous dissipation terms should be retained in situations where either fluid has high viscosity coefficient or its average velocity is high. Our second goal is to furnish series solutions for velocity and temperature by optimal homotopy method using package BVPh 2.0. Averaged squared residuals for the system are worked out that support the series solutions obtained. The results indicate that contribution of elasticity combined with viscous dissipation term is significant in the analysis of resisting torque, wall shear, entrained flow and heat transfer rate.

## Problem formulation

**Figure 1:**
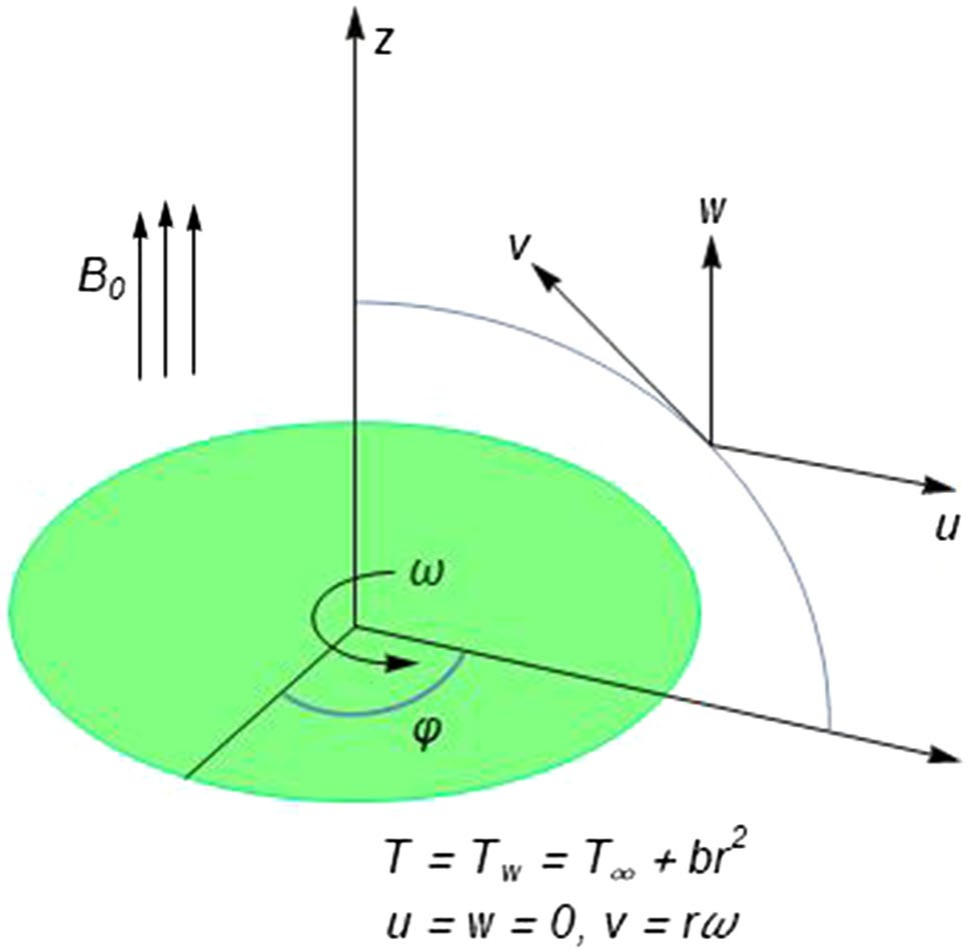
Geometry of the problem and coordinate system.

Suppose that an electrically conducting elastico-viscous fluid flows due to steady rotation of an infinite plane surface. Fluid is exposed to axial magnetic field with uniform magnetic flux density $B_{0}$. In a cylindrical coordinate system $\left( {r,\varphi ,z} \right)$, the disk taken along $z = 0$ is made to rotate steadily about the axis $r = 0$ (see Fig. 1). Fluid motion takes place in the semi-infinite region $z \ge 0$ and $z = 0$ is the only boundary. Let $u,v$ and $w$ symbolize velocity vector projections along $r - ,\varphi -$ and $z$-directions respectively. Assuming that electric field is absent and magnetic Reynolds number is small enough so that induced magnetic field is negligible, components of Lorentz force vector are $F_{r} = - \sigma B_{0}^{2} u$, $F_{\varphi } = - \sigma B_{0}^{2} v$ and $F_{z} = 0$, where $\sigma$ is the fluid electrical conductivity. Since the problem is symmetric about the vertical axis, one can neglect variation in velocities in $\varphi$-direction, that is, $\partial /\partial \varphi \equiv 0$. Relevant equations embodying fluid flow about a rotating disk are^24^:

1 $$\frac{\partial u}{{\partial r}} + \frac{u}{r} + \frac{\partial w}{{\partial z}} = 0,$$

2 $$\rho \left( {u\frac{\partial u}{{\partial r}} + w\frac{\partial u}{{\partial z}} - \frac{{v^{2} }}{r}} \right) = \frac{{\partial S_{rr} }}{\partial r} + \frac{{\partial S_{rz} }}{\partial z} + \frac{{S_{rr} - S_{\varphi \varphi } }}{r} - \sigma {\rm B}_{0}^{2} u,$$

3 $$\rho \left( {u\frac{\partial v}{{\partial r}} + w\frac{\partial v}{{\partial z}} + \frac{uv}{r}} \right) = \frac{{\partial S_{r\varphi } }}{\partial r} + \frac{{\partial S_{z\varphi } }}{\partial z} + \frac{{2S_{r\varphi } }}{r} - \sigma {\rm B}_{0}^{2} v,$$

4 $$\rho \left( {u\frac{\partial w}{{\partial r}} + w\frac{\partial w}{{\partial z}}} \right) = - \frac{\partial P}{{\partial z}} + \frac{{\partial S_{rz} }}{\partial r} + \frac{{\partial S_{zz} }}{\partial z} + \frac{{S_{rz} }}{r},$$

where $S_{ij} \left( {i,j = 1 - 3} \right)$ are components of stress tensor ${\mathbf{S}}$. Beard and Walters^38^ proposed the following stress tensor ${\mathbf{S}}$ for elastico-viscous liquids:

5 $${\mathbf{S}} = - P{\mathbf{I}} + \eta_{0} {\mathbf{A}}_{1} - \kappa_{0} \frac{{D{\mathbf{A}}_{1} }}{Dt},$$

where ${\mathbf{A}}_{1} = \nabla {\mathbf{v}} + (\nabla {\mathbf{v}})^{{\varvec{t}}}$ is known as first Rivlin-Ericksen tensor, $\eta_{0}$ stands for apparent viscosity, $\kappa_{0}$ is termed material fluid parameter, $P$ stands for pressure, ${\mathbf{I}}$ is the identity tensor, and $D{\mathbf{A}}_{1} /Dt$ is the upper-convected time derivative defined below:

6 $$\frac{{D{\mathbf{A}}_{1} }}{Dt} = \frac{{d{\mathbf{A}}_{1} }}{dt} - \left( {\nabla {\mathbf{v}}} \right){\mathbf{A}}_{1} - {\mathbf{A}}_{1} (\nabla {\mathbf{v}})^{{\varvec{t}}} .$$

Above equations are to be solved for the following constraints:

7a $${\text{at}} z = 0:{ }u = 0, v = r\omega , w = 0,$$

7b $${\text{as}} z \to \infty : u \to 0, v \to 0.$$

The components of stress tensor ${\mathbf{S}}$ are obtained from Eq. (5) as follows:

8 $$S_{rr} = - P + 2\eta_{0} \frac{\partial u}{{\partial r}} - 2\kappa_{0} \left\{ {u\frac{{\partial^{2} u}}{{\partial r^{2} }} + w\frac{{\partial^{2} u}}{\partial r\partial z} - 2\left( {\frac{\partial u}{{\partial r}}} \right)^{2} - \frac{\partial u}{{\partial z}}\left( {\frac{\partial u}{{\partial z}} + \frac{\partial w}{{\partial r}}} \right)} \right\},$$

9 $$S_{\varphi \varphi } = - P + 2\eta_{0} \frac{u}{r} - 2\kappa_{0} \left\{ {\frac{u}{r}\frac{\partial u}{{\partial r}} - 3\frac{{u^{2} }}{{r^{2} }} + \frac{w}{r}\frac{\partial u}{{\partial z}} - \left( {\frac{\partial v}{{\partial z}}} \right)^{2} - \left( {\frac{\partial v}{{\partial r}} - \frac{v}{r}} \right)^{2} } \right\} ,$$

10 $$S_{zz} = - P + 2\eta_{0} \frac{\partial w}{{\partial z}} - 2\kappa_{0} \left\{ {u\frac{{\partial^{2} w}}{\partial r\partial z} + w\frac{{\partial^{2} w}}{{\partial z^{2} }} - \frac{\partial w}{{\partial r}}\left( {\frac{\partial u}{{\partial z}} + \frac{\partial w}{{\partial r}}} \right) - 2\left( {\frac{\partial w}{{\partial z}}} \right)^{2} } \right\},$$

11 $$S_{r\varphi } = \eta_{0} \left( {\frac{\partial v}{{\partial r}} - \frac{v}{r}} \right) - \kappa_{0} \left\{ {u\frac{{\partial^{2} v}}{{\partial r^{2} }} - \left( {3\frac{\partial u}{{\partial r}} + 2\frac{u}{r} + w\frac{\partial }{\partial z}} \right)\left( {\frac{\partial v}{{\partial r}} - \frac{v}{r}} \right) - \frac{\partial v}{{\partial z}}\left( {2\frac{\partial u}{{\partial z}} + \frac{\partial w}{{\partial r}}} \right)} \right\},$$

12 $$S_{\varphi z} = \eta_{0} \frac{\partial v}{{\partial z}} - \kappa_{0} \left[ {u\frac{{\partial^{2} v}}{\partial r\partial z} + w\frac{{\partial^{2} v}}{{\partial z^{2} }} - \left( {\frac{\partial v}{{\partial r}} - \frac{v}{r}} \right)\left( {\frac{\partial u}{{\partial z}} + 2\frac{\partial w}{{\partial r}}} \right) - \frac{u}{r}\frac{\partial v}{{\partial z}} - 3\frac{\partial v}{{\partial z}}\frac{\partial w}{{\partial z}}} \right],$$

13 $$S_{rz} = \eta_{0} \left( {\frac{\partial u}{{\partial z}} + \frac{\partial w}{{\partial r}}} \right) - \kappa_{0} \left[ {\left( {u\frac{\partial }{\partial r} + w\frac{\partial }{\partial z}} \right)\left( {\frac{\partial u}{{\partial z}} + \frac{\partial w}{{\partial r}}} \right) - \frac{\partial u}{{\partial r}}\frac{\partial u}{{\partial z}} - \frac{\partial w}{{\partial r}}\frac{\partial w}{{\partial z}} - 3\left( {\frac{\partial u}{{\partial z}}\frac{\partial w}{{\partial z}} + \frac{\partial u}{{\partial r}}\frac{\partial w}{{\partial r}}} \right)} \right].$$

Accounting Eqs. (8)–(13) and the boundary layer approximations, Eqs. (2) and (3) become:

14 $$u\frac{\partial u}{{\partial r}} + w\frac{\partial u}{{\partial z}} - \frac{{v^{2} }}{r} = \frac{{\eta_{0} }}{\rho }\frac{{\partial^{2} u}}{{\partial z^{2} }} - \frac{{\kappa_{0} }}{\rho }\left\{ {\begin{array}{*{20}c} {u\frac{{\partial^{3} u}}{{\partial r\partial z^{2} }} + w\frac{{\partial^{3} u}}{{\partial z^{3} }} - 4\frac{\partial u}{{\partial z}}\frac{{\partial^{2} u}}{\partial r\partial z}} \\ { - \frac{\partial u}{{\partial r}}\frac{{\partial^{2} u}}{{\partial z^{2} }} - 3\frac{\partial u}{{\partial z}}\frac{{\partial^{2} w}}{{\partial z^{2} }} - 2\frac{\partial w}{{\partial z}}\frac{{\partial^{2} u}}{{\partial z^{2} }}} \\ { - \frac{2}{r}\left( {\frac{\partial u}{{\partial z}}} \right)^{2} + \frac{2}{r}\left( {\frac{\partial v}{{\partial z}}} \right)^{2} } \\ \end{array} } \right\} - \frac{{\sigma {\rm B}_{0}^{2} }}{\rho }u,$$

15 $$u\frac{\partial v}{{\partial r}} + w\frac{\partial v}{{\partial z}} + \frac{uv}{r} = \frac{{\eta_{0} }}{\rho }\frac{{\partial^{2} v}}{{\partial z^{2} }} - \frac{{\kappa_{0} }}{\rho }\left\{ {\begin{array}{*{20}c} {u\frac{{\partial^{3} v}}{{\partial r\partial z^{2} }} + w\frac{{\partial^{3} v}}{{\partial z^{3} }} - 2\frac{\partial v}{{\partial z}}\frac{{\partial^{2} u}}{\partial r\partial z}} \\ { - 2\frac{\partial w}{{\partial z}}\frac{{\partial^{2} v}}{{\partial z^{2} }} - 3\frac{\partial v}{{\partial z}}\frac{{\partial^{2} w}}{{\partial z^{2} }} - \frac{6}{r}\frac{\partial u}{{\partial z}}\frac{\partial v}{{\partial z}}} \\ { - \frac{{\partial^{2} u}}{{\partial z^{2} }}\left( {\frac{\partial v}{{\partial r}} - \frac{v}{r}} \right) - \frac{u}{r}\frac{{\partial^{2} v}}{{\partial z^{2} }}} \\ \end{array} } \right\} - \frac{{\sigma {\rm B}_{0}^{2} }}{\rho }v,$$

while Eq. (4) vanishes identically. For the solution of Eqs. (1), (14) and (15), we use the transformations:

16 $$u = r\omega F^{\prime}\left( \eta \right),{ }v = r\omega G\left( \eta \right),{ }w = - 2\sqrt {\omega \nu } F\left( \eta \right),$$

with $\eta = z \left( {\omega /\nu } \right)^{1/2}$ as similarity variable.

With the aid of Eq. (16), the mass balance Eq. (1) is fulfilled whereas Eqs. (14) and (15) give rise to the following ODEs:

17 $$F^{\prime\prime\prime} + G^{2} + 2FF^{\prime\prime} - F^{{\prime}2} - K\left( {4F^{\prime}F^{\prime\prime\prime} + 2G^{{\prime}2} - 2FF^{{{\prime\prime}{\prime\prime}}} } \right) - MF^{\prime} = 0,$$

18 $$G^{\prime\prime} - 2F^{\prime}G + 2FG^{\prime} - K\left( {4F^{\prime}G^{\prime\prime} - 2FG^{\prime\prime\prime} - 2F^{\prime\prime}G^{\prime}} \right) - MG = 0,$$

and boundary conditions (7a) and (7b) are transformed as follows:

19a $$F\left( 0 \right) = 0, F^{\prime}\left( 0 \right) = 0, G\left( 0 \right) = 1,$$

19b $$F^{\prime} \to 0, G \to 0 {\text{as}} \eta \to \infty ,$$

where $K = \kappa_{0} \omega /\eta_{0}$ is the elasticity parameter and $M = \sigma {\rm B}_{0}^{2} /\rho \omega$ is termed magnetic interaction parameter.

## Heat transfer analysis

The difference between surface temperature and that of the ambient fluid serves as driving potential for heat flow from the disk to the fluid. In absence of heat generation/absorption, energy equation can be expressed in the following form:

20 $$\rho C_{p} \left( {u\frac{\partial T}{{\partial r}} + w\frac{\partial T}{{\partial z}}} \right) = \kappa \left\{ {\frac{1}{r}\frac{\partial }{\partial r}\left( {r\frac{\partial T}{{\partial r}}} \right) + \frac{{\partial^{2} T}}{{\partial z^{2} }}} \right\} + {\Phi },$$

where $\kappa$ stands for fluid thermal conductivity, $C_{p}$ symbolizes specific heat capacity and ${\Phi }$ shows viscous dissipation term given by

21 $${\Phi } = S_{rr} \left( {\frac{\partial u}{{\partial r}}} \right) + S_{\varphi \varphi } \left( \frac{u}{r} \right) + S_{zz} \left( {\frac{\partial w}{{\partial z}}} \right) + S_{r\varphi } \left( {\frac{\partial v}{{\partial r}} - \frac{v}{r}} \right) + S_{\varphi z} \left( {\frac{\partial v}{{\partial z}}} \right) + S_{rz} \left( {\frac{\partial u}{{\partial z}} + \frac{\partial w}{{\partial r}}} \right).$$

Using (21) in (20) and then simplifying the resulting expression using boundary layer assumptions, one obtains:

22 $$u\frac{\partial T}{{\partial r}} + w\frac{\partial T}{{\partial z}} = \frac{\kappa }{{\rho C_{p} }}\frac{{\partial^{2} T}}{{\partial z^{2} }} + \frac{\eta_0 }{{\rho C_{p} }}\left\{ {\left( {\frac{\partial u}{{\partial z}}} \right)^{2} + \left( {\frac{\partial v}{{\partial z}}} \right)^{2} } \right\} - \frac{{\kappa_{0} }}{{\rho C_{p} }}\left\{ {\begin{array}{*{20}c} {u\frac{\partial u}{{\partial z}}\frac{{\partial^{2} u}}{\partial r\partial z} + w\frac{\partial u}{{\partial z}}\frac{{\partial^{2} u}}{{\partial z^{2} }} + u\frac{\partial v}{{\partial z}}\frac{{\partial^{2} v}}{\partial r\partial z} + 3\frac{\partial u}{{\partial r}}\left( {\frac{\partial v}{{\partial z}}} \right)^{2} } \\ { + 3\frac{u}{r}\left( {\frac{\partial u}{{\partial z}}} \right)^{2} + w\frac{\partial v}{{\partial z}}\frac{{\partial^{2} v}}{{\partial z^{2} }} - 3\frac{\partial u}{{\partial z}}\frac{\partial v}{{\partial z}}\left( {\frac{\partial v}{{\partial r}} - \frac{v}{r}} \right)} \\ \end{array} } \right\}.$$

We substitute $T = T_{\infty } + \left( {T_{w} - T_{\infty } } \right)\theta \left( \eta \right)$, where $\theta \left( \eta \right)$ is non-dimensional temperature and wall temperature $T_{w}$ has the form $T_{w} = T_{\infty } + br^{2}$, in which $b > 0$ is a constant. Equation (22) yields the following ODE:

23 $$\frac{1}{Pr}\theta^{\prime\prime} - 2F^{\prime}\theta + 2F\theta^{\prime} + Ec\left\{ {F^{^{\prime\prime}2} + G^{^{\prime}2} } \right\} - KEc\left\{ {\begin{array}{*{20}c} {4F^{\prime}\left( {F^{^{\prime\prime}2} + G^{^{\prime}2} } \right)} \\ { - 2F\left( {F^{\prime\prime}F^{\prime\prime\prime} + G^{\prime}G^{\prime\prime}} \right)} \\ \end{array} } \right\} = 0,$$

and boundary conditions for $\theta$ are given below:

24 $$\theta \left( 0 \right) = 1{\text{ and }}\theta \to 0 {\text{as}} \eta \to \infty .$$

In Eq. (23), $Pr = \eta_0 C_{p} /\kappa$ gives the Prandtl number an $Ec = \omega^{2} / bC_{p}$ defines the Eckert number.

## Skin friction coefficients, local Nusselt number and volumetric flow rate

In examining Von Kármán boundary layer, an important characteristic is the shear stress experienced at the disk. We define the radial and tangential skin friction coefficients as follows:

25 $$C_{fr} = \frac{{\left. {S_{rz} } \right|_{z = 0} }}{{\rho \left( {r\omega } \right)^{2} }}, C_{f\theta } = \frac{{\left. {S_{\varphi z} } \right|_{z = 0} }}{{\rho \left( {r\omega } \right)^{2} }}.$$

Upon utilizing Eq. (16) and boundary conditions (19a) in Eq. (25), one arrives at:

26 $$Re^{1/2} C_{fr} = F^{\prime\prime}\left( 0 \right), Re^{1/2} C_{f\theta } = G^{\prime}\left( 0 \right).$$

Another important concept is the Nusselt number defined as $Nu = - rk\left( {\partial T/\partial z} \right)_{z = 0} /{\Delta }T$. It can be expressed as:

27 $$Re^{ - 1/2} Nu = - \theta^{\prime}\left( 0 \right).$$

Entrainment velocity $w\left( \infty \right)$ can be used to determine the amount of fluid sucked towards the disk of radius $R$ as follows:

28 $$Q = \mathop \smallint \limits_{0}^{R} - w\left( \infty \right)2\pi rdr = 2\sqrt {\nu \omega } F\left( \infty \right)\pi R^{2} .$$

## Series solutions using optimal homotopy analysis method (OHAM)

An improved version of the well-known homotopy analysis method (HAM) was developed by Liao^39^ with an aim to tackle strongly non-linear problems. The concept was based on computing the best possible value of the so-called auxiliary parameter that eventually accelerates the convergence of HAM solutions. Afterwards, Liao et al.^40^ came up with a user-friendly MATHEMATICA package BVPh 2.0 based on the HAM, which is freely accessible online at http://numericaltank.sjtu.edu.cn/BVPh.htm. Using basic idea of HAM, the unknown functions $F,G$ and $\theta$ are expressed as under:

29 $$F\left( \eta \right) = \mathop \sum \limits_{k = 0}^{ + \infty } F_{k} \left( \eta \right), G\left( \eta \right) = \mathop \sum \limits_{k = 0}^{ + \infty } G_{k} \left( \eta \right), \theta \left( \eta \right) = \mathop \sum \limits_{k = 0}^{ + \infty } \theta_{k} \left( \eta \right),$$

in which $F_{k} ,G_{k}$ and $\theta_{k}$ can be obtained from by formulating $k$*th*-order deformation equations corresponding to Eqs. (17)–(19b), (23) and (24). The initial guesses of the system conforming with the so called *rule of solution expression* and the boundary conditions (19a), (19b) and (24) are chosen as:

30 $$F_{0} \left( \eta \right) = 0, G_{0} \left( \eta \right) = e^{ - \eta } , \theta_{0} \left( \eta \right) = e^{ - \eta } ,$$

and auxiliary linear operators ${\mathcal{L}}_{F} ,{\mathcal{L}}_{G} ,{\mathcal{L}}_{\theta }$ for the system of Eqs. (17), (18) and (23) are selected as follows:

31 $${\mathcal{L}}_{F} \equiv \frac{{\partial^{3} }}{{\partial \eta^{3} }} - \frac{\partial }{\partial \eta } , {\mathcal{L}}_{G} \equiv \frac{{\partial^{2} }}{{\partial \eta^{2} }} - 1, {\mathcal{L}}_{\theta } \equiv \frac{{\partial^{2} }}{{\partial \eta^{2} }} - 1.$$

Obviously, the above operators satisfy the following conditions:

32 $${\mathcal{L}}_{F} \left[ {c_{1} + c_{2} e^{ - \eta } + c_{3} e^{\eta } } \right] = 0,$$

33 $${\mathcal{L}}_{G} \left[ {c_{4} e^{ - \eta } + c_{5} e^{\eta } } \right] = 0,$$

34 $${\mathcal{L}}_{F} \left[ {c_{6} e^{ - \eta } + c_{7} e^{\eta } } \right] = 0,$$

where $c_{1} - c_{7}$ are unknown constants to be determined.

It is customary to mention that the resulting solutions by the HAM contain auxiliary parameters $c_{0}^{F} ,c_{0}^{G}$ and $c_{0}^{\theta }$ which play essential part in ensuring and accelerating convergence of solution. Here we are primarily interested to estimate optimal values of such parameters which correspond to minimum squared residual of the system. We begin by defining the total squared residual $E_{T,k}$ in the interval $\left[ {a,b} \right]$ as follows:

35 $$E_{T,k} = E_{F,k} + E_{G,k} + E_{\theta ,k} ,$$

where $E_{F,k}$,$E_{G,k}$ and $E_{\theta ,k}$ are average squared residuals of Eqs. (17), (18) and (23) defined as (see Liao^39^ for details):

36 $$E_{F,k} = \frac{1}{b - a}\mathop \smallint \limits_{a}^{b} \left( {{\mathcal{N}}_{F} \left[ {F_{k} \left( \eta \right),G_{k} \left( \eta \right)} \right]} \right)^{2} d\eta ,$$

37 $$E_{G,k} = \frac{1}{b - a}\mathop \smallint \limits_{a}^{b} \left( {{\mathcal{N}}_{G} \left[ {F_{k} \left( \eta \right),G_{k} \left( \eta \right)} \right]} \right)^{2} d\eta ,$$

38 $$E_{\theta ,k} = \frac{1}{b - a}\mathop \smallint \limits_{a}^{b} \left( {{\mathcal{N}}_{\theta } \left[ {F_{k} \left( \eta \right),G_{k} \left( \eta \right),\theta_{k} \left( \eta \right)} \right]} \right)^{2} d\eta ,$$

where ${\mathcal{N}}_{F}$,${\mathcal{N}}_{G}$ and ${\mathcal{N}}_{\theta }$ are the associated non-linear differential operators. The optimal values of $c_{0}^{F} ,c_{0}^{G}$ and $c_{0}^{\theta }$ are determined by command “GetOptiVar” of BVPh 2.0 (see^39^ for details).

## Results and discussion

**Figure 2:**
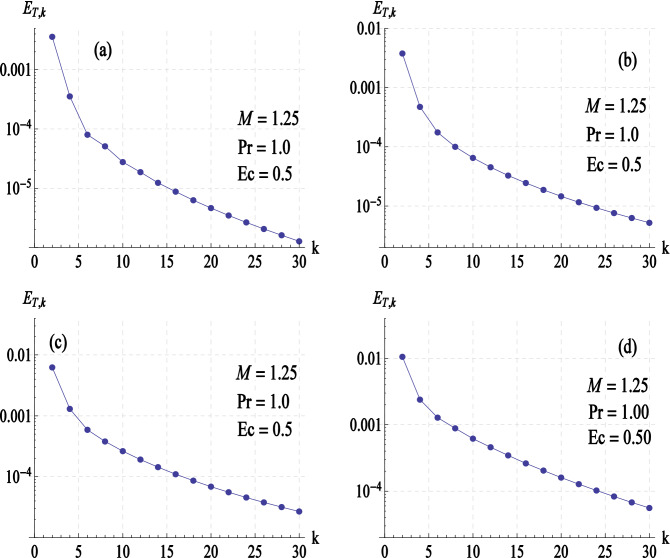
Total residual error (${\varvec{E}}_{{{\varvec{T}},{\varvec{k}}}}$) versus order of approximations (${\varvec{k}}$) at (**a**) ${\varvec{K}} = 0$, (**b**) ${\varvec{K}} = 0.25$, (**c**) ${\varvec{K}} = 0.5$ and (**d**) ${\varvec{K}} = 0.8$.

In order to ascertain that BVPh 2.0 code is working fine, we computed the total average squared residual (defined in Eqs. (35)) at different values of $K$, the elasticity parameter (see Fig. 2a–d). It is apparent that $E_{T,k}$ decreases monotonically as we increase $k$, the order of approximations. This confirms that series solutions given by Eq. (29) converge to the exact solutions as $k \to \infty$. For a further check, numerical results of $F^{\prime\prime}\left( 0 \right),G^{\prime}\left( 0 \right)$ and $\theta ^{\prime}\left( 0 \right)$ are compared with the numerical data of previous studies in limiting sense and found in complete agreement (see Table 1). Having validated the employed method, we now turn to foresee the role of different controlling parameters on the considered model.

**Table 1:** A comparison of 45th order HAM results with those obtained by Ariel^24,41^ for different values of $M$ when $K = 0$.

$M$	$F^{\prime\prime}\left( 0 \right)^{{\text{a}}}$	$- G^{^{\prime}} \left( 0 \right)^{{\text{a}}}$	$F^{\prime\prime}\left( 0 \right)^{{\text{b}}}$	$- G^{^{\prime}} \left( 0 \right)^{{\text{b}}}$
0.2	0.453141	0.708795	0.453129	0.708793
0.4	0.405576	0.802376	0.405576	0.802376
0.6	0.366698	0.894476	0.366698	0.894476
0.8	0.335092	0.983607	0.335090	0.983607
1.0	0.309258	1.069053	0.309258	1.069053
1.2	0.287915	1.150635	0.287915	1.150635
1.4	0.270049	1.228466	0.270049	1.228466
1.6	0.254892	1.302793	0.254892	1.302793
2.0	0.230559	1.442094	0.230559	1.442094

^a^Shows results given in Ariel^41^.

^b^Our results.

**Figure 3:**
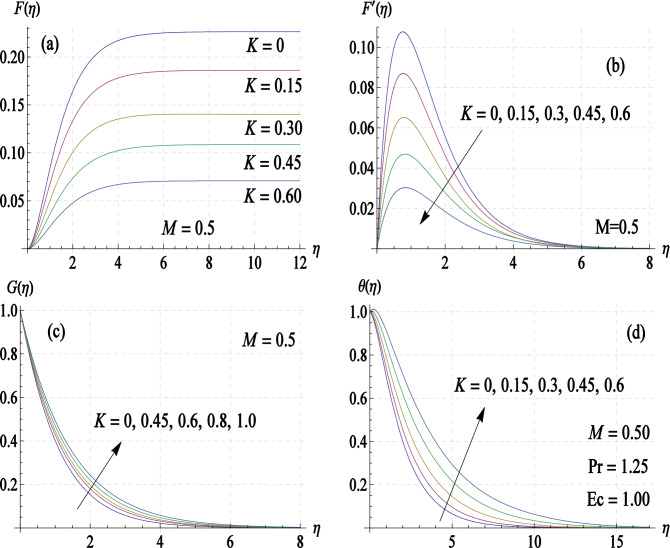
Curves of velocity components $\left( {{\varvec{F}},\user2{F^{\prime}},{\varvec{G}}} \right)$ and temperature $\left( {\varvec{\theta}} \right)$ for different values of elasticity parameter ${\varvec{K}}$.

The disk surface temperature is assumed to vary quadratically with radial distance $r$. Such an assumption is necessary for the governing problem to exhibit self-similar solutions. Figure 3a–d include velocity curves and temperature profile for varying choices of elasticity parameter $K$. Note that radial velocity $\left( {u = r\omega F^{\prime}} \right)$ and entrained flow are linked in such a way that the radially outflow, produced by centrifugal force, is responsible for drawing the fluid downwards towards the disk. Boundary layer is substantially thinned for increasing $K$-values. Reduction in radially driven flow by increasing $K$ is noted in Fig. 3a. This in turn leads to decelerate the axial fluid motion and thus the volumetric flow rate. No overshoot in similarity profiles is detected for the considered range of $K$. Circumferential flow is also predicted to slow down whenever elastic effects are considered (see Fig. 3c). On the contrary, fluid temperature rises for increasing $K$-values.

**Figure 4:**
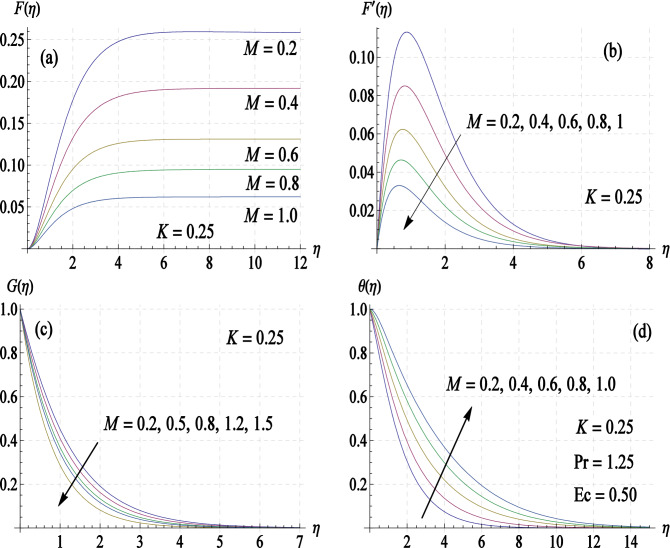
Curves of velocity components $\left( {{\varvec{F}},\user2{F^{\prime}},{\varvec{G}}} \right)$ and temperature $\left( {\varvec{\theta}} \right)$ for different values of magnetic interaction parameter ${\varvec{M}}$.

To see how present flow model is influenced by the presence of magnetic field, we prepared Fig. 4a–d showing velocity and temperature curves for a variety of $M$-values. It is noted that asymptotic value of $F$, that is $F\left( \infty \right)$, decreases for increasing values of $M$. Also, it takes shorter distances from the disk for the velocity profiles to attain their respective asymptotic values as $M$ is increased. Moreover, $u$-velocity profile $\left( {u = r\omega F^{\prime}} \right)$ becomes flatter for higher values of $M$. Furthermore, the resistance offered to fluid motion by the Lorentz force leads to enhancement in temperature profile as apparent from Fig. 4d.

**Figure 5:**
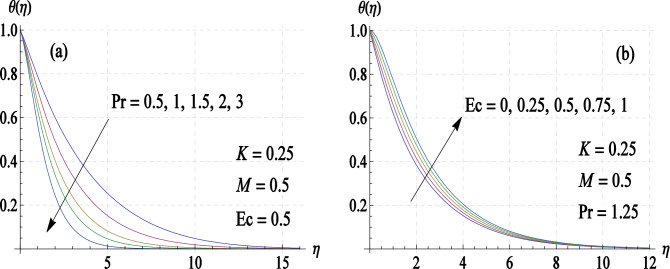
Temperature curves for different values of (**a**) Prandtl number $Pr$ and (**b**) Eckert number $Ec$.

Figure 5a shows the change in temperature distribution by varying $Pr$, the Prandtl number. It takes smaller distances from the disk for temperature curve to reach $\eta$-axis for increasing $Pr$-values. Moreover, the effect of Eckert number $Ec$ is seen to be typical of fluid gaining temperature (due to the loss of heat energy from the disk) (see Fig. 5b).

**Figure 6:**
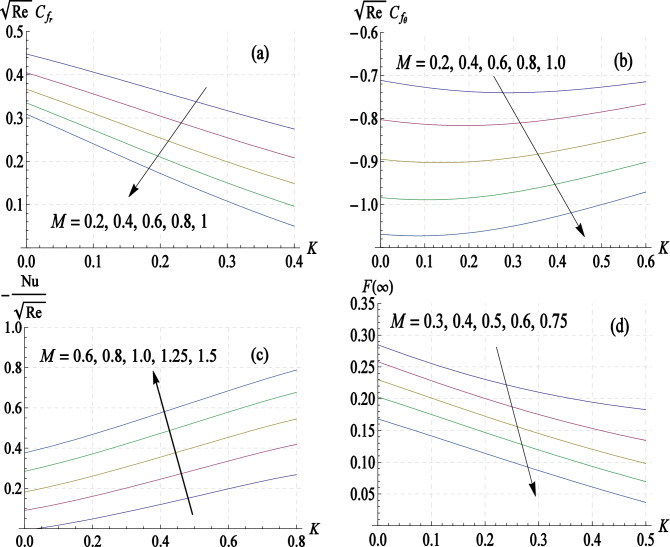
Profiles of skin friction coefficients, Nusselt number and volume flow rate versus elasticity parameter $K$ at different values of $M$.

In Fig. 6a–d, we present the graphs of $Re^{1/2} C_{{f_{r} }}$,$Re^{1/2} C_{{f_{\theta } }}$,$Re^{ - 1/2} Nu$ and $F\left( \infty \right)$ against the elasticity parameter $K$ for the values of latter in the range 0 to 1. While the results reveal that radial skin friction can be lowered by including elastic effects, the azimuthal skin friction first decreases to a minimum and then increases as $K$ increases. Nusselt number, measuring heat transfer rate, is predicted to elevate whenever $K$ enlarges. Interestingly, $F\left( \infty \right)$ has an inversely linear profile against both $K$ and $M$. Both radial and azimuthal wall stresses exhibit increasing trends for increasing values of $K$.

Table 2 contains the numerical data of entrainment velocity, radial wall stress and tangential wall stresses by changing the values of $K$ and $M$. Axial velocity at infinity, measuring the volume of entrained fluid is lowered whenever $K$ or $M$ is incremented. This reduction signals a growth in velocity gradients at the surface which yields higher magnitude of skin friction coefficients. Hence, we conclude that larger torque at the disk is required whenever elasticity and magnetic field effects are present.

**Table 2:** Computational results of skin friction coefficients at different values of $K$ and $M$ at 20th-order of approximations.

$K$	$M$	$F\left( \infty \right)$	$Re^{\frac{1}{2}} C_{{f_{r} }}$	$Re^{\frac{1}{2}} C_{{f_{\theta } }}$
0.2	0.5	0.175165	0.279125	− 0.857647
0.3		0.147619	0.226650	− 0.850164
0.4		0.122389	0.178885	− 0.835553
0.5		0.097984	0.136315	− 0.816793
0.2	0.2	0.295671	0.368621	− 0.728808
	0.5	0.175165	0.279125	− 0.857647
	0.8	0.104394	0.209732	− 0.984472
	1.2	0.052581	0.139514	− 1.144154

Numerical data exhibiting the effect of involved physical parameters on Nusselt number is tabulated (see Table 3). For higher $Pr$-values, heat convection measuring heat transfer rate from the surface is significant relative to pure conduction. Hence, Nusselt number increases in absolute sense for increasing values of $Pr$. Heat dissipation due to fluid friction strengthens as $Ec$ becomes large. This in turn yields expansion in temperature profile and reduction in Nusselt number. Figure 2a already indicated a clear reduction in axial velocity whenever elastico-viscous fluid is considered. Thus magnitude of the term $w\partial T/\partial z$ (in Eq. (9)), measuring heat convection, reduces when $K$ is enhanced. As a result, Nusselt number is seen to lower substantially when $K$ enlarges. Similar conclusion can be made for the influence of magnetic force on Nusselt number.

**Table 3:** Results of Nusselt number at different values of $K,M,Pr$ and $Ec$ at 20th-order of approximations.

$K$	$M$	$Pr$	$Ec$	$- \theta^{^{\prime}} \left( 0 \right)$
0	0.5	1.25	0.5	0.295399
0.2				0.228719
0.4				0.145372
0.6				0.057209
0.2	0.2			0.380507
	0.4			0.280761
	0.6			0.176379
	1.0			0.008257
	0.5	0.5		0.167541
		0.75		0.193142
		1.00		0.213086
		1.25		0.228719
		1.25	0	0.451032
			0.30	0.316919
			0.70	0.139779
			1.00	0.006359

## Concluding remarks

In this framework, we discussed elastico-viscous fluid flow bounded by a rotating disk with heat dissipation effects. The analysis is based on a quadratic surface temperature distribution which is a prerequisite for achieving self-similar solution. The developed system of equations is treated via package BVPh 2.0 of MATHEMATICA based on the HAM. The specific conclusions of the present study are outlined as follows:

- Using the package BVPh 2.0, the averaged squared residual of the governing system is computed which reflects that series solutions converge to the exact solutions as $k$ (order of approximation) tends to infinity.
- Akin to earlier works (see, for instance^23,26^), an increase in elasticity parameter $K$ has an retarding effect on the boundary layer flow. The entrained volume of the fluid upon the disk also decreases with an enhancement in elasticity.
- The effect of elasticity is such that radial wall stress decreases as values of $K$ are incremented. However, the resisting torque first decreases to a minimum and then increases for increasing $K$-values. Notably, for sufficiently higher values of $M$, resisting torque is monotonically increasing function of $K$.
- An expansion in thermal boundary layer is found for increasing values of $K$. Such increase accompanies with reduced heat transfer rate from the rotating surface.
- The existence of axial magnetic field opposes radially outward flow initiated by the centrifugal force. Such opposition restricts the amount of fluid drawn vertically thereby providing an expansion in temperature profile.
- As we increase the Eckert number, a relative decrease in enthalpy is noticed which in turn leads to an enhancement in the temperature profile.
